# Inbred Strain-Specific Response to Biglycan Deficiency in the Cortical Bone of C57BL6/129 and C3H/He Mice

**DOI:** 10.1359/jbmr.081259

**Published:** 2008-12-29

**Authors:** Joseph M Wallace, Kurtulus Golcuk, Michael D Morris, David H Kohn

**Affiliations:** 1 Department of Biomedical Engineering, The University of Michigan Ann Arbor, Michigan, USA; 2 Department of Chemistry, The University of Michigan Ann Arbor, Michigan, USA; 3 Department of Biologic and Materials Sciences, The University of Michigan Ann Arbor, Michigan, USA

**Keywords:** inbred mice, mechanical properties, μCT, PCR, Raman, histomorphometry

## Abstract

Inbred strain-specific differences in mice exist in bone cross-sectional geometry, mechanical properties, and indices of bone formation. Inbred strain-specific responses to external stimuli also exist, but the role of background strain in response to genetic deletion is not fully understood. Biglycan (bgn) deficiency impacts bone through negative regulation of osteoblasts, resulting in extracellular matrix alterations and decreased mechanical properties. Because osteoblasts from C3H/He (C3H) mice are inherently more active versus osteoblasts from other inbred strains, and the bones of C3H mice are less responsive to other insults, it was hypothesized that C3H mice would be relatively more resistant to changes associated with bgn deficiency compared with C57BL6/129 (B6;129) mice. Changes in mRNA expression, tissue composition, mineral density, bone formation rate, cross-sectional geometry, and mechanical properties were studied at 8 and 11 wk of age in the tibias of male wildtype and bgn-deficient mice bred on B6;129 and C3H background strains. Bgn deficiency altered collagen cross-linking and gene expression and the amount and composition of mineral in vivo. In bgn's absence, changes in collagen were independent of mouse strain. Bgn-deficiency increased the amount of mineral in both strains, but changes in mineral composition, cross-sectional geometry, and mechanical properties were dependent on genetic background. Bgn deficiency influenced the amount and composition of bone in mice from both strains at 8 wk, but C3H mice were better able to maintain properties close to wildtype (WT) levels. By 11 wk, most properties from C3H knockout (KO) bones were equal to or greater than WT levels, whereas phenotypic differences persisted in B6;129 KO mice. This is the first study into mouse strain-specific changes in a small leucine-rich proteoglycan gene disruption model in properties across the bone hierarchy and is also one of the first to relate these changes to mechanical competence. This study supports the importance of genetic factors in determining the response to a gene deletion and defines biglycan's importance to collagen and mineral composition in vivo.

## INTRODUCTION

Mice with targeted mutations in bone matrix proteins have been used to study the proteins' roles in regulating bone matrix deposition, composition, and mechanical integrity and aid in understanding how these functions relate to bone disease and fracture etiology.(1) One such model of disrupted protein production is the biglycan (bgn)-deficient mouse.(2–4) Bgn is a small leucine-rich proteoglycan (SLRP) that is enriched in the extracellular matrix (ECM) of bone and other connective tissues.(5–7) Bgn-deficient mice exhibit a defect in the growth and differentiation of osteoblasts resulting in decreased bone production and function.(8–10) Eleven-week-old bgn-deficient male mice exhibited decreased tissue-level yield strength, a property that is independent of the amount of tissue present.(11) Decreased tissue-level strength therefore suggests that deficiencies in bone ECM quality are responsible, a notion supported by data showing that the diameter of collagen fibrils in bgn-deficient bone is larger and more variable than in wildtype mice and often exhibit notches, protuberances, and irregular spacing.(3,4) Bgn-deficient bones also have greater volumetric BMD (vBMD)(11) and larger mineral crystal size(12) compared with wildtype (WT) mice. However, the same inbred strain of mouse was not consistently used to analyze these ECM changes. Because the genetic background of mice is known to influence skeletal properties, mice from different inbred strains may also respond differentially to a genetic deletion.

The C3H/He (C3H) and C57BL6 (B6) inbred strains have been used as models of high and low BMD, respectively.(13) Inbred strain-specific differences exist in bone cross-sectional geometric properties,(14,15) mechanical properties,(14,17) and indices of bone formation.(18,21) Inbred strain-specific differences also exist in response to external stimuli including mechanical loading and unloading,(22–24) bone regeneration after injury,(25,26) and new bone induction at an extraskeletal site,(27) and consistently show that C3H mice have a less intense response to changes in their mechanical environment than other inbred strains. The hybrid C57BL6/129 (B6;129) strain has been used as the background strain for many genetic knockouts (KO), including bgn deficiency.(2,11) In the tibias of male mice at 3 mo of age, vBMD is similar in B6(28) and B6;129 mice,(11) suggesting that the B6;129 mouse is an appropriate low bone mass strain to use in comparison with the high bone mass C3H mouse.

Understanding how genetic background influences the response to a gene deletion (in this case biglycan) is necessary to accurately infer the roles that biglycan plays in vivo. The effect of genetic background is consistently overlooked when studying the skeletal effects of a gene deletion and has never been studied in the genetic deletion of an SLRP. Biglycan (and bgn deficiency) is known to impact bone growth and bone mass in mice. When this genetic deletion is superimposed onto genetic backgrounds encoding for high and low bone mass/density, the resulting skeletal effects could greatly differ. By determining the phenotypic differences in mice from different inbred strains, novel insight into how biglycan influences collagen and mineral amount and composition, and ultimately bone mechanical function, can be obtained (e.g., how genetic modifiers modulate the functional effects of the gene). Because of the broad genetic variation in humans, specifically in BMD and fracture risk, this type of study may also help to extend the insight gained on the function of bgn in genotypes of high and low BMD to human populations. Bgn deficiency impacts the bone matrix through negative regulation of osteoblast number and function,(8–10) resulting in ECM alterations(3,4,11,12) and decreased mechanical properties.(11) Osteoblasts from C3H mice are more highly active and robust versus osteoblasts from other mouse strains, and the bones of C3H mice are less responsive to insult. It was therefore hypothesized that, compared with the response in B6;129 mice, C3H mice would be relatively more resistant to altered collagen structure and mineral composition associated with bgn deficiency. Therefore, the negative mechanical effects of bgn deficiency would be reduced in C3H mice relative to the effects in B6;129 mice. Because the bgn-deficient phenotype is strongest in the male tibia,(11) changes mRNA expression (qRT-PCR), tissue composition (Raman microspectroscopy), bone formation (dynamic histomorphometry), cross-sectional geometry and vBMD (μCT), and mechanical properties (four-point bending) were studied in the tibias of 8- and 11-wk-old male WT and bgn-deficient mice bred on B6;129 and C3H backgrounds to uncover inbred strain-specific responses to bgn deficiency across multiple levels of the bone hierarchy and to link phenotypic changes to functional competence.

## MATERIALS AND METHODS

### Animals

Procedures were performed at the University of Michigan with University Committee on Use and Care of Animals (UCUCA) approval (protocol 8518). Biglycan-deficient (KO) and WT breeder mice were the generous gift of Dr. Marian F. Young (National Institute of Dental and Craniofacial Research). KO mice from the B6;129 background strain were generated by homologous recombination in embryonic stem cells(2) and were backcrossed to the C3H/HeNHsd (C3H) strain to a purity of >95%.(9) On arrival at the University of Michigan, genotypes were verified using DNA extracted by tail biopsy and repeated for the F_1_ generation as secondary verification. Separate breeder lines were used to generate KO mice (by KO × KO matings) and WT mice (by WT × WT matings) in each background strain.

To determine proper sample sizes for detecting effects of genotype and background strain, power calculations were performed based on published values for differences and SDs in mechanical and geometric properties between inbred mouse strains(15,17) and because of bgn deficiency in B6;129 male mice(11) using a value of α = 0.05 and a power of 0.80. To statistically detect inbred strain specificity in response to the gene deletion, further power calculations were carried out based on expected ratios (KO/WT) between the two inbred strains.(29) To statistically detect inbred strain-specific responses and the effects of genotype and background strain in primary outcome measures (vBMD, cross-sectional size and shape, strength and deformation at whole bone and tissue levels), a sample size of *n* = 15 was used (2 inbred strains × 2 ages × 2 genotypes × *n* = 120 mice).

Mice were weaned at 3 wk of age and maintained in standard cages with access to food, water, and cage activity ad libitum. At 8 wk (day 0), mice from each background strain/genotype were randomly assigned to one of two weight-matched groups. Groups were killed at 8 and 11 wk. Mice in the 11-wk groups were given intraperitoneal injections of calcein (15 mg/kg body mass on day 4) and xylenol orange (80 mg/kg body mass on day 9). Mice were killed by CO_2_ inhalation, body mass was measured, and left tibias were harvested, stripped of soft tissue, wrapped in gauze soaked in a calcium-buffered saline solution, and stored at −20°C.

### μCT

Left tibias were scanned by μCT at 18 μm/voxel resolution (GE/EVS MS-8 specimen scanner; GE Healthcare, London, Ontario, Canada) and 3D images were reconstructed. Each 3D dataset was arranged as a series of 18-μm-thick slices oriented along the long axis of the tibia. Tibial length was measured directly on each reconstructed image from the most proximal portion of the condyles to the most distal portion of the medial malleolus (MicroView version 2.1.2; GE Healthcare).

A standard site in the diaphysis of each bone was located 792 μm proximal to the location where the tibia and fibula become fused (TFJ), a site chosen to lie just distal to the mechanical testing region (which began 800 μm proximal to the TFJ). Cross-sectional geometric properties and vBMD were determined from six consecutive μCT slices centered at this location. vBMD was determined using a threshold level of 2000 (MicroView version 2.1.2; GE Healthcare). For the measurement of geometric properties, each section was separated into bone and nonbone voxels using a previously defined method.(30) Geometric properties for each section were determined using a custom analysis program (total cross-sectional area, cortical area, marrow area (the difference between total area and cortical area), anterior-posterior (AP) and medial-lateral (ML) width, bending moment of inertia about the AP and ML axes (I_AP_, I_ML_), and average cortical thickness).

### Mechanical testing

Left tibias were brought to room temperature before testing and kept hydrated in calcium-buffered saline until the test was complete. Bones were tested in the ML direction (medial surface in tension) in four-point bending (Admet eXpert 450 Universal Testing Machine; Norwood, MA, USA). The fibula was carefully removed from each bone using a scalpel, and the bones were positioned with the TFJ aligned with the outside edge of one loading roller, preloaded to 0.5 N, preconditioned for 15 s (2 Hz, mean load of 2 ± 2 N), and monotonically tested to failure in displacement control at a rate of 0.025 mm/s. Load and deflection were recorded, from which structural strength (yield and ultimate forces), stiffness (slope of the linear portion of the force versus displacement curve), and deformation (yield deformation, postyield deformation, and total deformation) were determined.(11,31)

Bones were visually monitored during testing, and the point of fracture initiation was measured relative to the proximal end. A subset of geometric properties at the fracture site was obtained from μCT data (I_AP_ and the distance from the centroid to the tensile surface of the bone, c). Together with the load and deflection data, I_AP_ and c were used to map force and displacement (structural-level properties dependent on bone structural organization) into stress and strain (predicted tissue-level properties) from standard beam-bending equations for four-point bending:


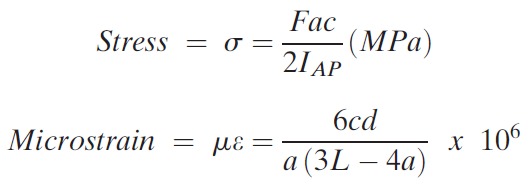


In these equations, *F* is the force, *d* is the displacement, *a* is the distance from the support to the inner loading point (3 mm), and *L* is the span between the outer supports (9 mm). The yield point was calculated using the 0.2% offset method based on the stress-strain curve. The modulus of elasticity was calculated as the slope of the linear portion of the stress-strain curve.

### Histomorphometry

After fracture testing, the distal half of all bones was dehydrated in graded ethanol (70%, 80%, 95%, 100%), defatted in Clear-Rite 3 (Richard-Allen Scientific, Kalamazoo, MI, USA), and infiltrated in a liquid methylmethacrylate monomer (Koldmount; Mager Scientific). The bones were embedded in poly methylmethacrylate (Koldmount Cold Mounting Kit; Mager Scientific). Using a low-speed sectioning saw (Model 650; South Bay Technology, San Clemente, CA, USA) with a diamond wafering blade (Mager Scientific), sections 100–150 μm thick were made from 11-wk-old bones. These sections were hand ground and polished to a thickness between 50 and 75 μm using wet silicon carbide abrasive discs (sections were located an average distance of 570 ± 397 μm proximal to the TFJ). Sections were imaged at a magnification of ×200 (Nikon Eclipse TE 300) using the Nikon FITC-TRITC-DAPI filter combination (Calcein [FITC] excitation at 475–490 nm, emission at 505–535 nm; Xylenol Orange [TRITC] excitation at 545–565 nm, emission at 580–620 nm) and analyzed using digital analysis software (ImageJ, version 1.36b). Histomorphometric analyses were performed using standard ASBMR methods and nomenclature.(32) Bone surface lengths (BS), labeled surfaces (single label [sL]; double label [dL]), and center-to-center interlabel distances (Ir.L.Th) were measured on both the endocortical and periosteal surfaces (Es., Ps.). The time between each injection (Ir.L.t) was 5 days. Mineralizing surface (MS), mineral apposition rate (MAR), and bone formation rate (BFR) were determined at each surface:


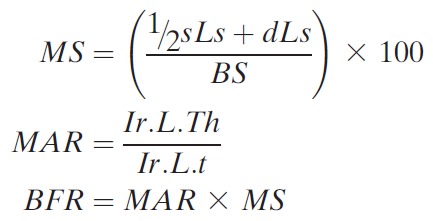


### Raman microspectroscopy

Six bones from each group were randomly chosen for analysis by Raman spectroscopy (11-wk bones were used after sectioning for histomorphometry). Thick sections (≥3 mm) were made and hand polished using wet silicon carbide abrasive discs (the average location of this section was 510 ± 262 μm distal to the TFJ).

The Raman system used in this study has been described.(33) Briefly, Raman scatter was excited using a 785-nm laser with a rectangular beam profile (Kaiser Optical Systems) and passed through a ×20 objective that focuses the line-shaped beam (∼100 μm in length). Raman scattered light from every point on the line was simultaneously passed back through the objective and through a dichroic mirror to a charge coupled device detector.

Band areas were determined: a phosphate band (*PO*_4_^3−^ν_1_ symmetric stretch, 957 cm^−1^), a carbonate band (*CO*_4_^2−^ν_1_ symmetric stretch, 1070 cm^−1^), and the amide I band (C=O stretch, 1595–1720 cm^−1^). The amide I band was decomposed into two smaller bands at 1660 and 1690 cm^−1^. The mineral/matrix ratio was determined by dividing the phosphate band area by the amide I band area. The carbonate/phosphate ratio (indicative of carbonate substituting in the crystal lattice for phosphate ions) was determined by dividing the carbonate band area by the phosphate band area. Mineral crystallinity (indicative of the size, shape, and perfection of mineral crystals) was obtained from the inverse of the full bandwidth at one-half peak intensity of the phosphate band. The collagen cross-linking ratio (indicative of the amount of nonreducible/reducible cross-linking) was determined by dividing the 1660-cm^−1^ band area by the 1690-cm^−1^ band area.(34) Twelve spectral lines were collected from each sample (periosteal, intracortical, and endocortical locations in each anatomic quadrant). Because values were not statistically different between regions, the 12 values were pooled to obtain an overall measurement for each specimen.

### Real-time quantitative RT-PCR

Right tibias from 11-wk mice were pulverized and stored in RNA-STAT60 (IsoTex Diagnostics) after death. Within each group, equal numbers of the 15 bones were randomly pooled into three samples (five bones in each). RNA was isolated from each pooled sample and cleaned of DNA (Qiagen RNase-Free DNase Set), and cDNA was synthesized (SuperScript II kit; Invitrogen). One pooled sample from each experimental group (e.g., B6;129 11-wk WT) was randomly chosen for analysis, with the other two samples from each group serving as biological replicates.

Real-time qRT-PCR was performed (ABI 7500 PCR System; Applied Biosystems). Following manufacturer's protocols (ABI Prism 7700 Sequence Detection System, User Bulletin 2), mRNA expression levels for each sample/primer were normalized to rRNA 18S levels and expressed as a fold change relative to the background-strain WT level. All primer/probe mixtures were TaqMan Gene Expression Assays (Applied Biosystems). In lieu of primer/probe sequences, ABI Assay ID numbers are given: decorin (dcn; Mm00514535_m1), fibromodulin (fm; Mm00491215_m1), type I procollagen α1 (Col1a1; Mm00801666_g1), type I procollagen α2 (Col1a2; Mm00483888_m1), and TGF-β1 (Mm00441724_m1). The selected genes of interest were chosen to study the regulation of other SLRPs by bgn (DCN and FM) because of data suggesting altered SLRP expression in the absence of bgn,(10) alterations in type I collagen expression (Col1a1 and Col1a2) because of known fibril changes in bgn-deficient mice,(3,4) and changes in the expression of a growth factor that is known to be responsive to bgn.(35) As an endogenous control, rRNA 18S was used (ABI assay 4352930E).

### Statistical analysis

All statistical analyses used Sigma Stat (Version 3.1; Systat Software) or SPSS (Version 11.0; SPSS). To determine whether inbred strain-specific differences in the bgn-deficient phenotype existed in each property at each age, ratios of the KO/mean WT value for each KO sample within each age and inbred strain were calculated. The difference in ratios between inbred strains at each age was analyzed using Student's *t*-tests looking for the effects of background strain and genotype. To detect phenotypic effects (KO versus WT) within each background strain/age, Student's *t*-tests were also used. In groups that failed to exhibit normal distributions or equal variance, Mann-Whitney rank sum tests were performed. Linear regressions were performed to determine relationships between body mass (as the independent variable) and all geometric, mechanical and compositional properties. Normalization to body mass was restricted to those properties that were found to have a significant relationship with body mass (i.e., cross-sectional size and preyield structural-level mechanical properties). For all studies, a value of *p* < 0.05 was considered significant, whereas 0.05 < *p* < 0.10 was also noted. All data are shown as mean ± SD.

## RESULTS

### Body mass and tibial length

B6;129 KO mice had significantly decreased body mass (*p* = 0.048) and tibial length (*p* < 0.001) versus WT mice at 8 wk of age (Table 1). By 11 wk of age, body mass was no longer different in B6;129 KO mice versus WT mice, but tibial length was still significantly decreased (*p* < 0.001). The body mass of C3H KO mice was significantly greater than WT mice at 8 wk of age (*p* = 0.014), whereas there was no difference in tibial length. At 11 wk, body mass was still greater in C3H KO mice versus WT mice (*p* = 0.049), whereas tibial length still did not differ.

**Table 1:** Body Mass and Tibial Length (Mean ± SD)

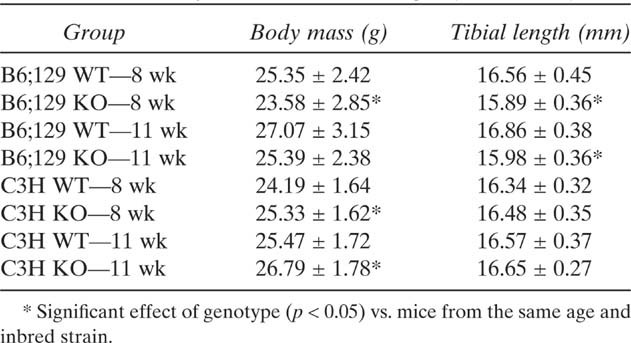

To study inbred strain specificity in response to bgn deficiency for body mass and tibial length, the relative effects of the gene deletion in B6;129 mice (ratio of KO/mean WT values for each KO sample) were statistically compared with the effects in C3H mice (ratio of KO/mean WT value). At 8 wk, B6;129 KO mice weighed less than their WT counterparts whereas the opposite was true in C3H mice, resulting in a significant inbred strain-specific body mass phenotype (*p* < 0.001). Inbred strain specificity also existed in tibial length at 8 wk (decreased KO/WT ratio in B6;129; *p* < 0.001). At 11 wk, significant inbred strain-specific phenotypic differences existed in both body mass (increased KO/WT ratio in C3H versus B6;129 mice; *p* < 0.001) and tibial length (increased ratio in B6;129 mice; *p* < 0.001).

### Tissue composition

**FIG. 1:**
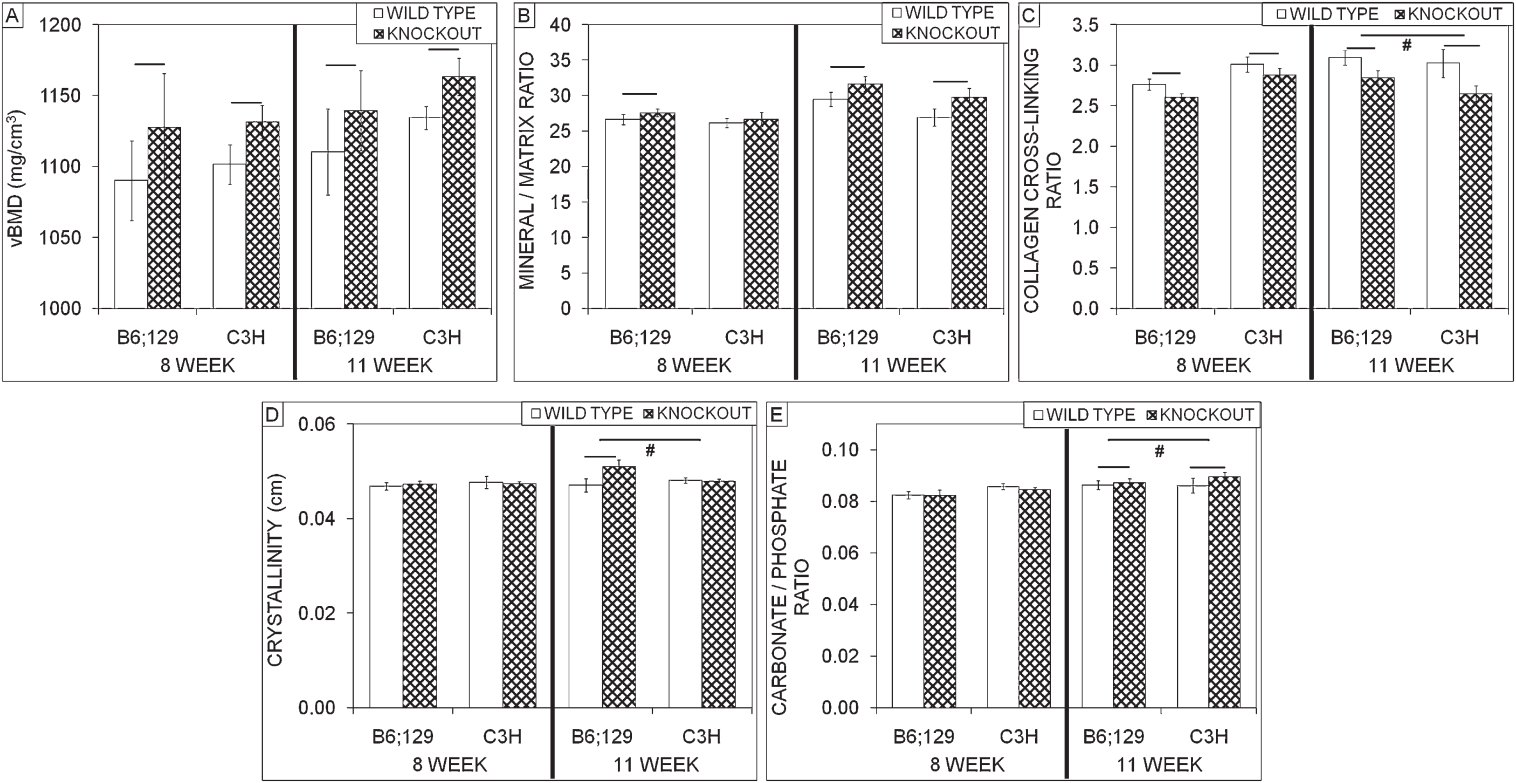
vBMD and tissue composition from the tibial mid-diaphysis of wildtype and bgn-deficient mice. Data are presented as mean ± SD. At 8 wk, B6;129 KO mice had significantly increased vBMD (A) and mineral/matrix ratio (B), but a decreased collagen cross-linking ratio (C) vs. WT mice (indicated by a line above the histograms). C3H KO mice had significantly increased vBMD but a decreased collagen cross-linking ratio vs. WT mice. Inbred strain specificity was inspected by comparing the difference in the ratio of KO/WT between inbred strains (*p* < 0.05 indicated by line with # below it), and was absent at 8 wk. At 11 wk, B6;129 KO mice had significantly increased vBMD, mineral/matrix ratio, and crystallinity (D), but a decreased collagen cross-linking ratio vs. WT mice. C3H KO mice had increased vBMD, mineral/matrix ratio and carbonate/phosphate ratio (E), but a decreased collagen cross-linking ratio vs. WT mice. Inbred strain specificity existed in the collagen cross-linking ratio, crystallinity, and the carbonate/phosphate ratio at 11 wk.

At 8 wk of age, B6;129 KO mice had significantly increased vBMD (*p* = 0.004) and mineral/matrix ratio (*p* = 0.028), whereas the collagen cross-linking ratio was significantly decreased (*p* < 0.001) versus WT mice (Fig. 1). At 11 wk, vBMD (*p* = 0.011), the mineral/matrix ratio (*p* = 0.004) and crystallinity (*p* < 0.001) were significantly greater in B6;129 KO mice versus WT mice, whereas the collagen cross-linking ratio was significantly decreased (*p* < 0.001). In C3H mice at 8 wk of age, vBMD was significantly greater in the KO mice versus WT mice (*p* < 0.001) and was accompanied by a significant decrease in the collagen cross-linking ratio (*p* = 0.031). At 11 wk, vBMD (*p* < 0.001), the mineral/matrix ratio (*p* = 0.003), and the carbonate/phosphate ratio (*p* = 0.028) were significantly greater in the C3H KO mice versus WT mice, whereas the collagen cross-linking ratio was significantly decreased (*p* < 0.001).

No inbred strain specificity existed in compositional properties at 8 wk of age. By 11 wk, inbred strain specificity was noted in the collagen cross-linking ratio (greater decrease in C3H KO versus WT than in B6;129 KO versus WT; *p* = 0.027; Fig. 1C, #), crystallinity (increased KO/WT ratio in B6;129; *p* = 0.002; Fig. 1D, #), and the carbonate/phosphate ratio (increased KO/WT ratio in C3H mice; *p* = 0.027; Fig. 1E, #).

### Bone cross-sectional geometry and histomorphometry

**FIG. 2:**
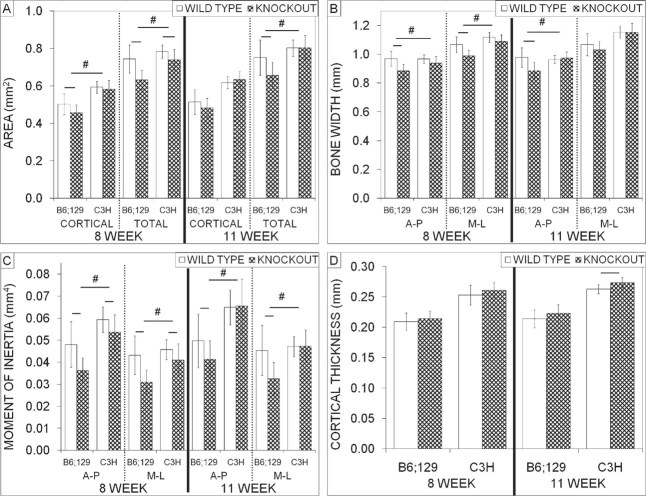
Cross-sectional geometric properties from the tibial mid-diaphysis of wildtype and bgn-deficient mice. Data are presented as mean ± SD. At 8 wk, all properties were significantly decreased in B6;129 KO mice vs. WT mice except cortical thickness (D), whereas in C3H KO mice, total area (A), marrow area, AP moment of inertia (MOI), and ML MOI (C) were significantly less than in WT mice (indicated by a line above the histograms). Inbred strain specificity (indicated by a line with # above it) was present in all properties except cortical thickness. At 11 wk, B6;129 KO mice had significantly smaller total area, marrow area, AP width (B), AP MOI, and ML MOI vs. WT mice. In C3H KO mice, cortical thickness was significantly greater than WT mice at 11 wk. Differences in total area, marrow area, AP width, AP MOI, and ML MOI were inbred strain specific.

**FIG. 3:**
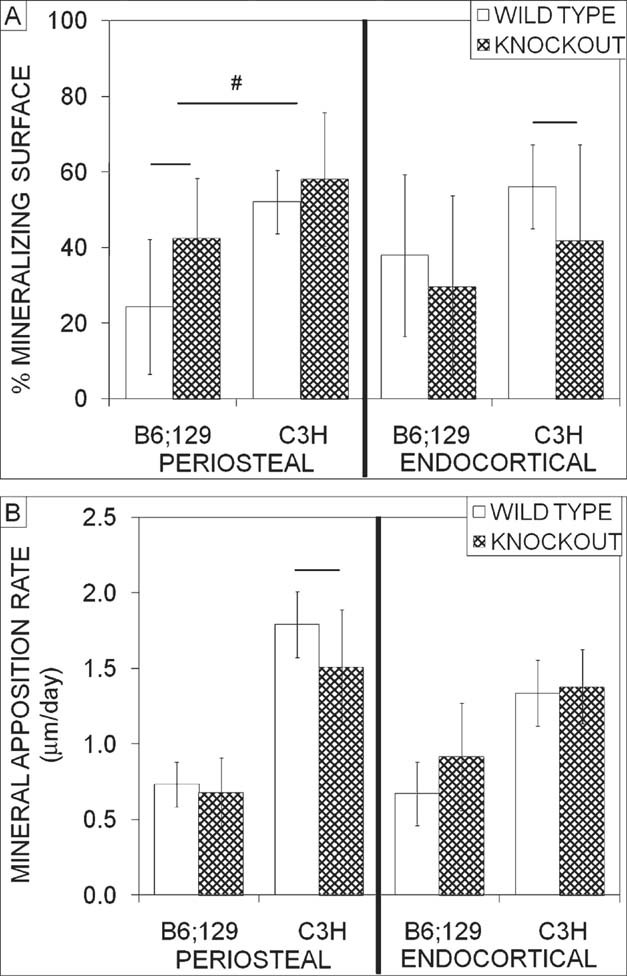
Histomorphometry from the tibial mid-diaphysis of wildtype and bgn-deficient mice between 8 and 11 wk of age. Data are presented as mean ± SD. B6;129 KO mice had significantly increased periosteal mineralizing surface (A) vs. WT mice (indicated by a line above the histograms). C3H KO mice had significantly decreased percent endocortical mineralizing surface and periosteal mineral apposition rate (B) vs. WT mice. The difference in percent periosteal mineralizing surface was inbred strain specific (indicated by a line with # above it).

B6;129 KO mice had significantly decreased total area (*p* < 0.001), cortical area (*p* = 0.018), marrow area (*p* < 0.001, data not shown), AP width (*p* < 0.001), ML width (*p* < 0.001), I_AP_ (*p* < 0.001), and I_ML_ (*p* < 0.001) versus WT mice at 8 wk of age (Fig. 2). At 11 wk of age, KO mice had significantly smaller total area (*p* = 0.004), marrow area (*p* < 0.001, data not shown), AP width (*p* < 0.001), AP/ML ratio (*p* < 0.001, data not shown), I_AP_ (*p* = 0.038), and I_ML_ (*p* = 0.005) versus WT. Between 8 and 11 wk of age, periosteal mineralizing surface was significantly elevated in the B6;129 KO mice versus WT mice (*p* = 0.006; Fig. 3A).

In C3H mice at 8 wk of age, total cross-sectional area (*p* = 0.015), marrow area (*p* < 0.001, data not shown), I_AP_ (*p* = 0.034), and I_ML_ (*p* = 0.044) were significantly less in the KO mice versus WT mice (Fig. 2). At 11 wk of age, cortical thickness was significantly greater in KO mice versus WT mice at (*p* = 0.001). Between 8 and 11 wk of age, endocortical mineralizing surface (*p* = 0.050; Fig. 3A) and periosteal mineral apposition rate (*p* = 0.019; Fig. 3B) were significantly decreased in the C3H KO mice versus WT mice.

Most cross-sectional geometric properties were decreased in KO mice from both inbred strains compared with WT levels at 8 wk of age, but the KO/WT ratios were significantly less in B6;129 versus C3H mice, indicating an inbred strain-specific decrease in total area (*p* = 0.002; Fig. 2A, #), cortical area (*p* = 0.029; Fig. 2A, #), marrow area (*p* = 0.007, data not shown), AP width (*p* = 0.003; Fig. 2B, #), ML width (*p* = 0.002; Fig. 2B, #), I_AP_ (*p* = 0.005; Fig. 2C, #), and I_ML_ (*p* = 0.003; Fig. 2C, #). Differences in total area (*p* < 0.001; Fig. 2A, #), marrow area (*p* < 0.001, data not shown), AP width (*p* < 0.001; Fig. 2B, #), AP/ML ratio (*p* < 0.001, data not shown), I_AP_ (*p* = 0.018; Fig. 2C, #), and I_ML_ (*p* < 0.001; Fig. 2C, #) were inbred strain specific because of significant decreases in B6;129 KO mice versus WT mice at 11 wk of age. Periosteal mineralizing surface showed an inbred strain-specific increase in B6;129 KO mice between 8 and 11 wk of age (*p* < 0.001; Fig. 3A, #).

### Tissue-level mechanical properties

**FIG. 4:**
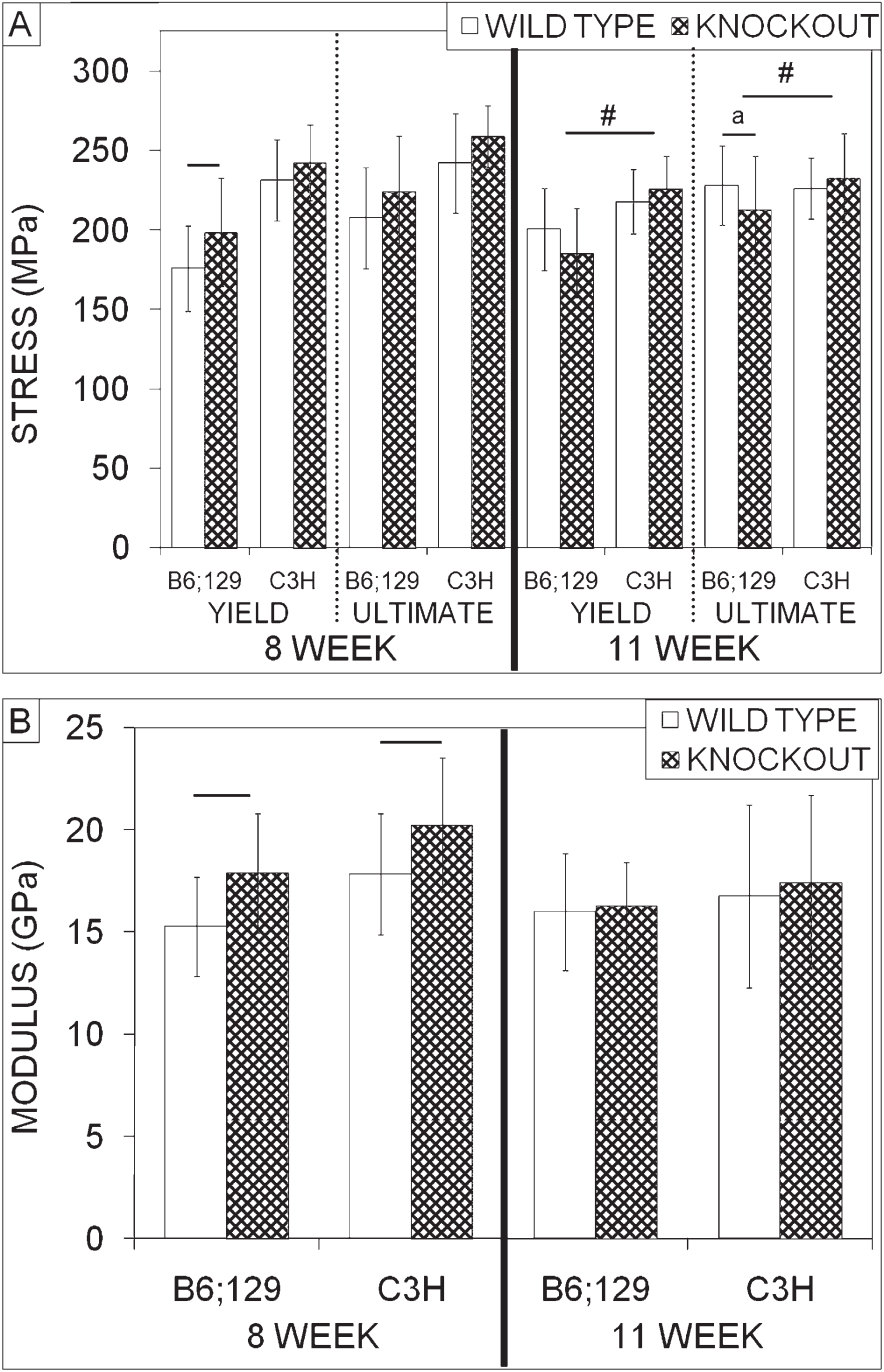
Estimated tissue-level mechanical properties from the tibial mid-diaphysis of wildtype and bgn-deficient mice. Data are presented as mean ± SD. In B6;129 KO mice at 8 wk, yield stress (A) and modulus (B) were significantly greater vs. WT mice, whereas in C3H KO, modulus was significantly greater vs. WT mice (indicated by a line above the histograms). Inbred strain specificity (indicated by a line with # above it) was absent at 8 wk. In B6;129 KO mice at 11 wk, ultimate stress (A) was marginally decreased vs. WT mice (*p* < 0.056, indicated by a line above the histograms with “a” above it). No tissue-level mechanical properties differed in C3H KO vs. WT mice at 11 wk. Inbred strain-specific differences existed in yield stress and ultimate stress.

In B6;129 mice at 8 wk of age, yield stress (*p* = 0.049) and modulus (*p* = 0.012) were significantly greater in KO versus WT mice (Fig. 4). By 11 wk, no properties were significantly different in KO mice versus WT mice, but ultimate stress (*p* = 0.056) was marginally decreased. In C3H mice at 8 wk of age, there was a significant increase in modulus in KO mice versus WT mice (*p* = 0.050). By 11 wk, no properties differed between C3H KO mice and WT mice.

At 8 wk of age, no inbred strain specificity existed in tissue-level mechanical properties, whereas at 11 wk, inbred strain-specific differences existed in yield and ultimate stress (decreased KO/WT ratio in B6;129; *p* = 0.018 and *p* = 0.050 respectively; Fig. 4A, #).

### Structural-level mechanical properties

**FIG. 5:**
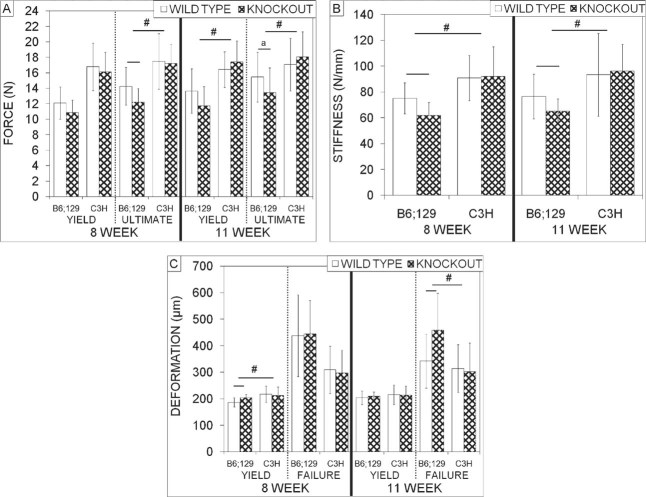
Structural-level mechanical properties from the tibial mid-diaphysis of wildtype and bgn-deficient mice. Data are presented as mean ± SD. At 8 wk, ultimate force (A) and stiffness (B) were significantly decreased in the B6;129 KO mice vs. WT mice, whereas yield deformation (C) was greater (indicated by a line above the histograms). No properties differed in C3H KO versus WT mice, so inbred strain specificity (indicated by a line with # above it) existed in ultimate force, stiffness, and yield deformation. At 11 wk, stiffness was significantly decreased, and ultimate force was marginally decreased (*p* < 0.053, indicated by a line above the histograms with “a” above it) in B6;129 KO mice vs. WT mice, whereas failure and postyield deformation (equal to the difference between failure and yield deformation) were significantly increased. No properties were different in C3H KO vs. WT mice at 11 wk, so inbred strain specificity existed in yield force, ultimate force, stiffness, and postyield and failure deformation.

At 8 wk of age in B6;129 mice, ultimate force (*p* = 0.006) and stiffness (*p* = 0.003) were significantly decreased in the KO versus WT mice, whereas yield deformation was significantly greater (*p* = 0.003; Fig. 5). Stiffness was significantly decreased (*p* = 0.034) and ultimate force was marginally decreased (*p* = 0.053) in KO mice versus WT mice at 11 wk, whereas failure deformation (*p* = 0.014) and postyield deformation (*p* = 0.016, equal to the difference between failure and yield deformation) were significantly increased. In C3H mice, no properties differed between KO and WT mice at either 8 or 11 wk of age.

At 8 wk of age, inbred strain-specific differences existed in ultimate force (decreased KO/WT ratio in B6;129; *p* = 0.013; Fig. 5A, #), stiffness (decreased ratio in B6;129; *p* = 0.024; Fig. 5B, #), and yield deformation (increased ratio in B6;129; *p* = 0.019; Fig. 5C, #). At 11 wk, inbred strain-specific phenotypic differences existed in yield force (decreased KO/WT ratio in B6;129; *p* = 0.002; Fig. 5A, #), ultimate force (decreased ratio in B6;129 mice; *p* = 0.005; Fig. 5A, #), stiffness (decreased ratio in B6;129 mice; *p* = 0.007; Fig. 5B, #), failure deformation (increased ratio in B6;129 mice; *p* = 0.011; Fig. 5C, #), and postyield deformation (increased ratio in B6;129 mice; *p* = 0.023, data not shown).

### mRNA expression at 11 wk of age

**FIG. 6:**
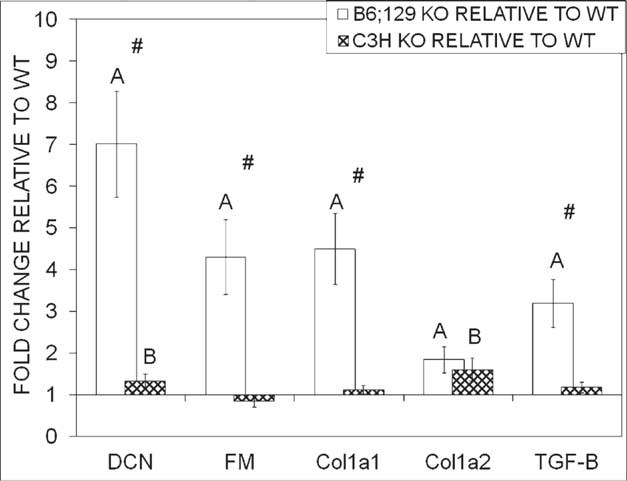
mRNA expression from the right tibias of wildtype and bgn-deficient mice at 11 wk of age. Data are presented as mean ± SD. mRNA expression levels for decorin (DCN), fibromodulin (FM), procollagen1α1 (Col1a1), procollagen1α2 (Col1a2), and TGFβ were significantly upregulated in B6;129 KO mice relative to WT levels (indicated by A). C3H KO mice had significantly elevated expression of DCN and Col1a2 relative to WT levels (indicated by B). Inbred strain specificity (indicated by #) was present in the expression of DCN, FM, Col1a1, and TGFβ.

mRNA expression levels for all genes of interest were significantly increased (*p* < 0.05) in B6;129 KO mice relative to WT levels at 11 wk (Fig. 6). In C3H mice, mRNA expression levels for DCN (*p* = 0.005) and Col1a2 (*p* = 0.029) were significantly upregulated in KO mice. Phenotypic differences in the expression of DCN (*p* < 0.001), FM (*p* = 0.029), Col1a1 (*p* < 0.001), and TGF-β (*p* < 0.001) were inbred strain specific at 11 wk of age (ratio of [gene of interest/18S in KO]/[gene of interest/18S in WT] increased in B6;129 versus the ratio in C3H mice for all of these genes; Fig. 6).

### Effects of body mass

The effect of body mass on the bgn-deficient phenotype in mice from different background strains is an important consideration (Table 2). Linear regressions were therefore performed between body mass (as the independent variable) and all other properties measured in this study. Significant relationships with body mass (all positive) were only noted in cross-sectional geometric parameters and structural strength (yield and ultimate) and stiffness (Table 2; we also show tissue-level properties that lacked a significant correlation with body mass for comparison). Further analyses about effects of body mass were therefore restricted to cross-sectional size and preyield structural-level properties.

**Table 2:** Linear Regressions With Body Mass

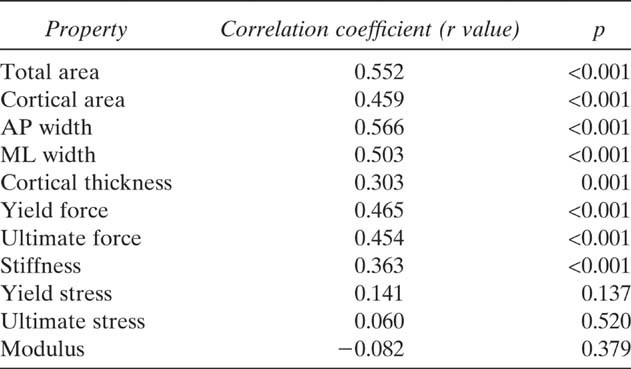

At 8 wk of age, on normalization to body mass, inbred strain-specific deficiencies in geometric properties in B6;129 mice were absent. However, deficiencies in C3H KO mice were magnified, with AP and ML widths now showing inbred strain-specific decreases in C3H mice. There were no longer inbred strain-specific differences in structural-level mechanical properties in either inbred strain.

At 11 wk of age, most inbred strain-specific deficiencies in cross-sectional geometric properties in B6;129 KO mice were maintained on normalization to body mass (except total area and AP width). Despite these cross-sectional deficiencies, inbred strain-specific deficiencies in structural-level strength (yield and ultimate force) and stiffness in B6;129 mice were now absent on normalization to body mass.

## DISCUSSION

At 8 wk of age, the bgn-deficient phenotype was more significant in B6;129 mice versus C3H mice, resulting in an inbred strain-specific response to this genetic change (significant difference in KO/WT ratio in B6;129 versus C3H) in body mass, tibial length, multiple cross-sectional geometric properties, and structural stiffness and strength. The relationship between inbred strain and bgn-deficient phenotype became more compelling by 11 wk of age, impacting the B6;129 mice more than the C3H mice at all levels of the bone hierarchy that were studied. At 11 wk, inbred strain-specific differences existed in body mass, tibial length, mRNA expression (SLRPs, type I collagen, TGF-β), tissue composition, tissue strength, cross-sectional geometric properties, and structural strength, stiffness, and ductility.

The bones of inbred mouse strains vary in properties ranging from cellular activity to cross-sectional geometry.(13–21) Bones from inbred mouse strains have shown inbred strain-specific responses to many experimental conditions, including mechanical loading and fracture healing.(22–26) Genetic differences in mice are also important in determining the response to genetic deletion.(36,37) To the best of our knowledge, this study represents the first detailed investigation into inbred strain-specific changes in bone in a murine SLRP gene disruption model. This is also one of the first studies to relate differences in mechanical competence to properties across the bone hierarchy ranging from the molecular and chemical makeup of the tissue through structural organization. This study supports the notion that the clinical severity of diseases in humans may be driven in part by genetic influence, specifically in human populations with varying BMD and fracture risk. Because of the weight placed on conclusions drawn from gene deletion studies, results from this study highlight the importance of taking into account variability that can arise from one inbred mouse strain to another in interpreting structure-function relations resulting from a gene deletion.

Using Raman microspectroscopy (and vBMD from μCT) allowed us to infer how the absence of bgn influenced bone composition in vivo, and how these changes were modulated by the genetic background of the mice. Bgn-deficient mice from both inbred strains had a decrease in the collagen cross-linking ratio versus WT mice (Fig. 1C), implying a change in the secondary structure of collagen most often associated with the ratio of mature to immature cross-links.(34) These mice also had increased procollagen mRNA expression (Fig. 6). Decreased collagen cross-linking and increased collagen gene expression are consistent with differences noted in the size, shape, and spacing of collagen fibrils in B6;129 KO mice versus WT mice.(3,4) Our data suggest that biglycan is necessary for the formation and organization of the organic matrix independent of background strain, extending our knowledge of the role of biglycan in in vivo function.

Bgn-deficient mice from both inbred strains had greater vBMD (Fig. 1A) and mineral/matrix ratio (Fig. 1B) versus WT mice, supporting bgn's role in also regulating mineralization in vivo.(11) However, B6;129 KO mice had increased crystallinity (Fig. 1D), whereas C3H KO mice had increased carbonate/phosphate ratio (Fig. 1E) versus WT mice. Therefore, the differences in mineral composition noted in KO mice from different background strains suggest that, whereas bgn regulates the relative amount of mineral present in the matrix, the composition and structure of the resulting mineral are dependent on the genetic background of the mice. As recently shown by Jepsen et al.(38) in regard to biological processes that co-adapt traits for mechanical function in different inbred strains, differences in mineral composition in bgn-deficient mice from different background strains in our study may be driven by different optimization strategies used by the inbred strains. Because estimated tissue-level mechanical properties (Fig. 4) are independent of the amount and distribution of tissue, inbred strain-specific differences in mechanical properties likely arose from these differences in tissue composition (Fig. 1).

Altered collagen and mineral in KO bones from both inbred strains suggest two possible scenarios. In the first, bgn deficiency causes direct changes in the composition and structure of the developing collagen matrix. Because collagen forms the template for mineralization, the mineral that forms on an altered matrix can be changed in both composition and density,(39) as supported by increased crystallinity in B6;129 KO mice (Fig. 1D) and increased carbonate/phosphate ratio in C3H KO mice (Fig. 1E). Mineral nucleates in the gap zones of collagen, and SLRPs localize within these zones.(7) It is therefore possible that nucleation sites that are normally blocked by bgn are exposed in the absence of bgn and more mineral can form, as supported by increased vBMD (Fig. 1A) and mineral/matrix ratio (Fig. 1B). In the second scenario, the lack of bgn directly impacts mineralization. Bgn might facilitate the initial nucleation of mineral, but further crystal growth in preferential directions may be blocked by the presence of bgn near a specific crystal face,(40,41) meaning that, in the absence of bgn, crystals can grow to larger than normal dimensions. Unrestricted crystal growth along specific planes could explain both the increase in vBMD (Fig. 1A) and increased crystallinity (Fig. 1D). As mineral beyond normal levels fills the spaces within collagen fibrils and the fibrils are distorted,(42) the dissociation/rupture of some cross-links may occur. This hypothesis is supported by transmission electron microscopy (TEM) data from WT and KO mice that indicate a greater variation in the mineral orientation and local mineral density in KO mice from both inbred strains (data not shown).

Body mass differences between KO and WT mice from each inbred strain raise the concern that body mass may be acting as a confounding factor in the analysis of inbred strain specificity. Cross-sectional geometric properties, yield, ultimate force, and stiffness had a significant relationship with body mass (Table 2), and these properties were normalized by body mass for further analysis. When normalized at 8 wk of age, inbred strain-specific deficiencies in geometric properties in B6;129 mice were absent, but deficiencies in C3H KO mice were magnified. There were no longer inbred strain-specific differences in structural-level mechanical properties in either inbred strain. When normalized at 11 wk of age, most inbred strain-specific deficiencies in cross-sectional geometric properties in B6;129 KO mice were maintained. Despite these cross-sectional deficiencies, inbred strain-specific deficiencies in structural-level strength and stiffness in B6;129 mice were now absent.

Any conclusions drawn from other properties in this study (gene expression, tissue composition, histomorphometry, tissue-level mechanical properties, and postyield structural-level mechanical properties) were unchanged when body weight differences were corrected for, which is expected because these properties are not dependent on body weight or body size. Postyield properties were still increased in B6;129 KO versus WT mice with no changes in C3H KO mice, suggesting that inbred strain-specific differences in tissue quality in B6;129 KO mice are responsible. Because inbred strain-specific increases in postyield structural level properties in B6;129 mice are maintained along with inbred strain-specific changes in gene expression, histomorphometry, tissue composition, and tissue-level mechanical properties, the main conclusions of inbred strain specificity drawn from this study remain true. Specifically, the deletion of biglycan resulted in altered gene expression and tissue composition in both inbred strains, but the background strain of the mice dictated how these properties impacted bone size and mechanical integrity. These inbred strain-specific changes are an important and novel observation and hold true when properties are normalized to body mass.

In conclusion, the above data showed that biglycan influences collagen cross-linking and gene expression and the amount and composition of mineral in bone in vivo. Changes in collagen observed here are dependent on the absence of biglycan, independent of inbred strain. Bgn regulates the amount of mineral present, but the genetic background of the mice dictates the composition of the resulting mineral in bgn-deficient mice. Bgn deficiency influenced the amount and composition of bone in mice from both inbred strains at 8 wk of age, but C3H mice were better able to maintain properties near WT levels. In terms of bone size and mechanical integrity, bgn-deficient C3H mice approach their WT counterparts by 11 wk of age, whereas phenotypic deficiencies persisted in the B6;129 KO mice versus WT mice. This study is the first investigation into inbred strain-specific changes in the bones of mice in a SLRP gene disruption mode. This is also one of the first studies to relate differences in mechanical competence to properties across the bone hierarchy ranging from the molecular and chemical makeup of the tissue through structural organization. This study further supports the importance of genetic factors in determining the response to a gene deletion, and suggests that the clinical severity of diseases in humans may be driven in part by genetic influences, especially in populations with differing BMD and fracture risk.
